# Protein intake and cardiovascular diseases: an umbrella review of systematic reviews for the evidence-based guideline on protein intake of the German Nutrition Society

**DOI:** 10.1007/s00394-025-03746-2

**Published:** 2025-08-13

**Authors:** Sarah Egert, Anna M. Amini, Lea Klug, Nicole Kalotai, Julia Haardt, Heiner Boeing, Anette E. Buyken, Anja Kroke, Stefan Lorkowski, Sandrine Louis, Katharina Nimptsch, Matthias B. Schulze, Lukas Schwingshackl, Roswitha Siener, Gabriele I. Stangl, Armin Zittermann, Bernhard Watzl, Sabine Ellinger

**Affiliations:** 1 Institute of Nutritional and Food Science, Nutritional Physiology, University of Bonn, Bonn, Germany; 2 German Nutrition Society, Bonn, Germany; 3 Department of Epidemiology (closed), German Institute of Human Nutrition Potsdam-Rehbruecke, Nuthetal, Germany; 4 Institute for Nutrition, Consumption and Health, Faculty of Natural Science, Paderborn University, Paderborn, Germany; 5 Department of Nutritional, Food and Consumer Science, Fulda University of Applied Sciences, Fulda, Germany; 6 Institute of Nutritional Sciences, Friedrich Schiller University Jena, Jena, Germany; 7 Department of Physiology and Biochemistry of Nutrition, Max Rubner-Institut, Karlsruhe, Germany; 8 Molecular Epidemiology Research Group, Max Delbrück Center for Molecular Medicine (MDC) in the Helmholtz Association, Berlin, Germany; 9 Department of Molecular Epidemiology, German Institute of Human Nutrition Potsdam-Rehbruecke, Nuthetal, Germany; 10 Institute of Nutritional Science, University of Potsdam, Potsdam, Germany; 11 Institute for Evidence in Medicine, Faculty of Medicine, Medical Center, University of Freiburg, Freiburg, Germany; 12 Department of Urology, University Stone Center, University Hospital Bonn, Bonn, Germany; 13 Institute of Agricultural and Nutritional Sciences, Martin Luther University Halle-Wittenberg, Halle (Saale), Germany; 14 Clinic for Thoracic and Cardiovascular Surgery, Herz- und Diabeteszentrum Nordrhein Westfalen, Ruhr University Bochum, Bad Oeynhausen, Germany

**Keywords:** Umbrella review, Protein intake, Cardiovascular diseases, Stroke, Coronary heart disease

## Abstract

**Purpose:**

This umbrella review aimed to investigate the evidence for an association of dietary intake of total protein as well as animal and plant protein with the incidence of coronary heart disease (CHD), stroke and total cardiovascular diseases (CVD).

**Methods:**

PubMed, Embase and Cochrane Database were systematically searched for systematic reviews (SRs) of prospective studies with or without meta-analysis (MA) published between January 2012 and April 2024. Methodological quality, outcome-specific certainty of evidence, and overall certainty of evidence were assessed using established tools and predefined criteria.

**Results:**

Ten SRs were considered eligible for the umbrella review; all were based on prospective cohort studies, and six conducted a MA. Dietary intakes of total, animal and plant protein were not associated with the risk of CHD or stroke. For CHD, the overall certainty of evidence for the absence of an association was “probable” for total, animal and plant protein. For stroke and total CVD, the overall certainty of evidence was rated as “possible” for the absence of an association with the intake of total protein and plant protein and insufficient for animal protein intake.

**Conclusion:**

Given that most SRs on dietary protein intake did not indicate an association, it seems that protein intake plays no major role in the development of CVD. This investigation was registered at PROSPERO as CRD42018082395.

**Supplementary Information:**

The online version contains supplementary material available at 10.1007/s00394-025-03746-2.

## Introduction

Cardiovascular diseases (CVD) are the leading cause of death worldwide and substantially contribute to loss of health and excess health system costs [1]. Primary prevention of CVD is therefore of high importance. Coronary heart disease (CHD) and stroke are among the most common forms of CVD. The causes of CVD are multiple, with hypertension, obesity, diabetes mellitus, and dyslipidaemia being major risk factors for the development of CVD [2]. One of the contributing factors is an unbalanced (“unhealthy”) Western dietary pattern characterised by a high consumption of energy-dense ultra-processed foods as well as of animal protein (processed meat), saturated fatty acids (SAFA), added sugar and sodium [3, 4]. By contrast, so-called “health-promoting” dietary patterns, including the Mediterranean and the DASH diet, are rich in natural or only minimally processed foods, fruits, vegetables and vegetable oils, and in plant-based protein sources (e.g., legumes). Such dietary patterns are also lower in sodium, SAFA, and added sugars; they are also higher in unsaturated fatty acids, dietary fibre, micronutrients, and phytochemicals and are more satiating than a typical Western diet [4, 5]. Therefore, adherence to a Mediterranean-style diet or to the DASH diet is associated with a reduced risk of CVD [6, 7].

The role of dietary proteins in causing or preventing CVD is controversially discussed. A systematic review and dose–response meta-analysis (MA) found no association between total protein or animal protein and CVD mortality, whereas plant protein was associated with a lower risk [8]. A potential contribution of plant protein may be mediated through CVD risk factors. For example, clinical trials found that the consumption of soya protein (containing isoflavones) beneficially affected CVD risk factors such as hypertension [9] and improved blood lipids [10] as well as glycaemic control [11]. In addition, a wide range of mechanistic studies suggested cardioprotective properties of milk-derived bioactive peptides, including antihypertensive, antithrombotic, hypolipidaemic and antidiabetic effects [12]. The anti-hypertensive effect of milk peptides was confirmed in randomised controlled trials (RCTs) [13]. In addition to peptides, certain amino acids have been linked with beneficial effects on blood pressure and CVD [14]. For example, a recent MA of RCTs on the effects of L-arginine demonstrated significant decreases in systolic and diastolic blood pressure [15]. In addition, recent data of the EPIC cohort study indicate an inverse association of dietary proline intake with the risk of ischaemic stroke. Among dietary sources, dairy products had the highest correlation with proline intake [16].

While SRs on protein intake and risks of CVD have been published [17–26], a standardised assessment of the epidemiological evidence is lacking. Thus, the present umbrella review addressed the level and certainty of evidence derived from SRs concerning whether dietary intake of protein, and proteins from plant and animal sources in general, can modify the risk of CVD, including CHD and stroke, in the adult population. Our umbrella review will contribute to the upcoming evidence-based guideline for protein intake of the German Nutrition Society considering the development of different pathologies and selected health-related outcomes.

## Methods

We conducted an umbrella review (PROSPERO: CRD42018082395) following the methodology published by Kroke et al. [27]. Systematic literature search, selection of SRs, data extraction and the assessment of methodological quality and outcome-specific certainty of evidence were conducted independently in pairs by four authors (AMA, NK, LK, JH). Any disagreements were resolved by consensus.

### Data sources and searches

Systematic literature searches were conducted in PubMed, Embase and Cochrane Database of Systematic Reviews for SRs with or without MA published between January 2012 and April 2024. January 2012 results from the decision to cover a 10-year period, i.e. the first database search was conducted in January 2022 and the last update in April 2024. The search strategies are presented in Supplementary Material 1. In addition, reference lists of the included SRs were screened.

### Selection of systematic reviews

Titles and/or abstracts of retrieved records were screened according to the pre-defined inclusion/exclusion criteria to identify potentially eligible publications. The full-texts of potentially relevant publications were assessed for eligibility. It was tolerated that some of the primary studies were incorporated more than once in different SRs. The overlap of the primary studies was documented and the percentage of overlap was assessed by calculating the corrected cover area according to Pieper et al. [28].

SRs were included if they met the following criteria: (i) evaluated the association between protein intake and CVD morbidity (total CVD, CHD and stroke) in adults, including older adults and recreational athletes (studies investigating the totality of CVD morbidity and mortality were tolerated), (ii) SRs with or without MA of prospective studies in humans, i.e. RCTs and prospective cohort studies (including case-cohort studies and nested case–control studies). Inclusion of case–control studies was tolerated if another study type was predominant, and (iii) SRs written in English or German and published between January 2012 and April 2024.

Exclusion criteria were as followed: (i) study populations exclusively consisting of children, pregnant and/or lactating women and/or top athletes, (ii) not investigating the specific effect/associations of protein intake, (iii) exclusively investigating CVD mortality, (iv) not investigating relevant protein-outcome pairs, (v) conference proceedings or abstracts, (vi) individual primary studies, (vii) SRs exclusively based on case–control studies and/or cross-sectional studies, and (viii) umbrella reviews.

### Data extraction

The following data from each included SR were extracted into a standardised form: surname of first author, year of publication, type of SR, study duration, study population, intervention/exposure(s), outcome(s), effect estimate(s) including 95% CI, *P*-value(s), and heterogeneity estimate(s). In case of missing data, corresponding authors were contacted.

### Assessment of methodological quality and outcome-specific certainty of evidence

The methodological quality of the retrieved SRs was assessed by a modified version of the “A Measurement Tool to Assess Systematic Reviews 2” (AMSTAR 2) tool [29] (Supplementary Material 3). This considers the risk of bias assessment, the quality of statistical analyses, reporting of results and transparency of potential sources of conflict of each SR. SRs were rated on a scale from high to critically low quality by considering critical and non-critical methodological weaknesses. SRs graded as “critically low” by AMSTAR 2 were excluded from the current analysis. Methodological quality was assessed separately for each SR included.

The outcome-specific certainty of evidence of included SRs was assessed using the NutriGrade scoring tool [30]. Based on a numerical scoring system, four categories rate the outcome-specific certainty of evidence: high, moderate, low, and very low (Supplementary Material 4). The NutriGrade scoring tool was modified for the assessment of SRs without MA with the adaptions described by Kroke et al. [27]. If a SR reported more than one relevant outcome, each outcome-specific certainty of evidence was assessed separately.

The results of the SR quality analyses and the outcome-specific certainty of evidence were documented systematically (Supplementary Materials 5 and 6).

### Grading the overall certainty of evidence

Three authors (SE, SEg, LK) graded the overall certainty of evidence for each relevant exposure-outcome combination according to the criteria outlined in our protocol [27] and in Table 1. Briefly, the overall rating ranges from convincing, probable, possible to insufficient. First, we assessed whether there is at least one SR with or without MA of prospective studies. If more than one SR with or without MA was available, all (convincing) or the majority (probable, possible) of the results must be consistent. Biological plausibility must be given in any case (direct or inverse association). Finally, the results of the AMSTAR 2 and NutriGrade ratings were considered. Depending on the level of evidence, the SRs must have achieved a certain rating in both tools. If no SR was identified, or if the majority of SRs reached a very low outcome-specific certainty of evidence and/or low methodological quality, the overall certainty of evidence was considered insufficient. This rating was double-checked by an independent author (AMA) and was thereafter reviewed by all co-authors. The final ratings of the overall certainty of evidence were approved by all authors.

**Table 1:** Grading the overall certainty of evidence according to methodological quality, outcome-specific certainty of evidence, biological plausibility and consistency of results, and definition of the overall certainty of evidence in a modified form according to the GRADE approach [1, 12]

Overall certainty of evidence	Underlying criteria	Definition/explanation
Convincing	At least one SR with or without MA of prospective studies available If more than one SR with or without MA are available: all overall results must be consistent.^1^ In case of a positive or negative association, biological plausibility is given All included SRs with or without MA must reach at least a “moderate” outcome-specific certainty of evidence^2^; in addition all included SRs must reach at least a methodological quality^3^ of “moderate”	There is high level of confidence that the true effect lies close to that of the estimate(s) of the effect
Probable	At least one SR with or without MA of prospective studies available If more than one SR with or without MA are available, the majority of overall results must be consistent.^1^ In case of a positive or negative association, biological plausibility is given The majority^4^ of included SRs with or without MA must have reached at least a “moderate” outcome-specific certainty of evidence^2^; in addition all included SRs must reach at least a methodological quality^3^ of “moderate”	There is moderate confidence in the effect estimate(s): The true effect is likely to be close to the estimate of the effect, but there is a possibility that it is substantially different
Possible	At least one SR with or without MA of prospective studies available If more than one SR with or without MA are available, the majority of overall results must be consistent.^1^ In case of a positive or negative association, biological plausibility is given The majority^4^ of included SRs with or without MA must reach at least a “low” outcome-specific certainty of evidence^2^; in addition the majority^4^ of all included SRs must reach at least a methodological quality^3^ of “moderate”	Confidence in the effect estimate(s) is limited: The true effect may be substantially different from the estimate of the effect
Insufficient	No SR is available *OR* The majority^4^ of included SRs with or without MA reach a “very low” outcome-specific certainty of evidence^2^; in addition the majority of all included SRs reach a methodological quality^3^ of “low”	There is very little confidence in the effect estimate (s): The true effect is likely to be substantially different from the estimate of effect

^1^Consistent = overall results of the SR have to be consistently either risk reducing or risk elevating or consistently showing no risk association

^2^Outcome-specific certainty of evidence refers to the NutriGrade rating

^3^Methodological quality refers the AMSTAR 2 rating; SRs graded as “critically low” by AMSTAR 2 are not considered

^4^Majority: > 50% of the included SRs,

MA, meta-analysis; SR, systematic review

## Results

**Fig. 1:**
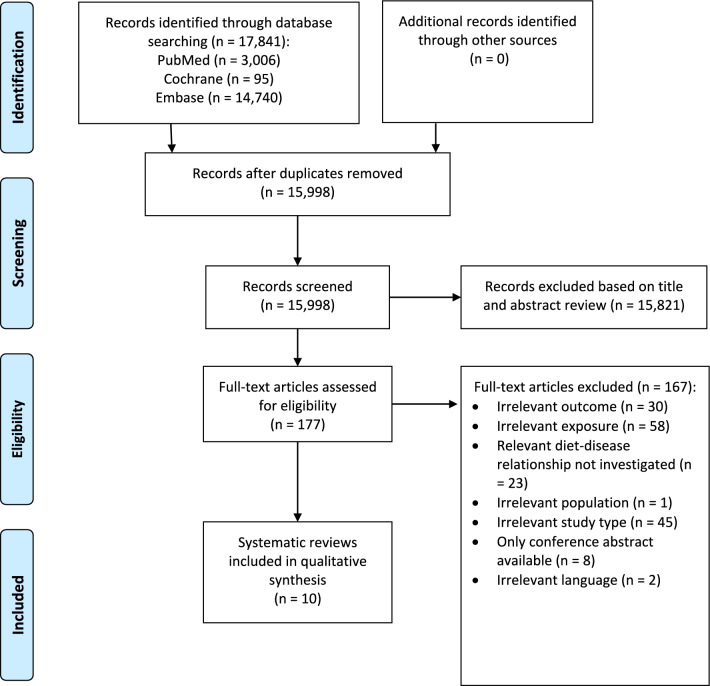
Flow diagram

The study selection process is outlined in Fig. 1. The literature search identified 17,841 potentially relevant records. After removing of 1,843 duplicates, 15,821 records were excluded on the basis of the title and/or abstract. Thereafter, 167 records were excluded after assessing the full-text. Finally, a total of ten SRs was included in the present umbrella review [17–26]. These SRs were published between July 2013 and January 2024. A list of excluded records after assessing the full-texts, including justifications for exclusion, is provided as Supplementary Material 7.

### Study characteristics

All ten SRs were exclusively based on prospective cohort studies [17–26]. Overall, the SRs included 34 primary studies, although the SR by Ma et al. 2024 [23] did not specify which primary studies were included in the analysis. In total, six SRs conducted a MA [18–21, 23, 25], and four SRs were without MA [17, 22, 24, 26]. Three SRs with MA analysed dose–response-relationships [18, 21, 23]. The included SRs investigated the following outcomes: CHD (5 SRs [17, 21, 22, 24, 26]), stroke (6 SRs [17–19, 22, 23, 25]), and total CVD (4 SRs [20, 22, 23, 25]). Some of the primary studies were incorporated more than once into different SRs, but overall, there was only a small overlap of primary studies of 9%, determined as corrected covered area according to Pieper et al. [28] (Supplementary Material 2).

Most SRs investigated the amount of total as well as plant and animal protein intake [18–23]. Three SRs investigated the intake of plant protein [20, 24, 26], two of them considered soya protein exclusively [20, 26]. Mantzouranis et al. [25] investigated the impact of high protein diets on CVD outcomes without differentiation between plant and animal protein.

In six evaluations from three SRs, the data on protein intake were given in percentage of total daily energy [22, 24, 25]. Only one evaluation reported the intake as absolute amount [26].

The follow-up of the included cohort studies ranged from 18 months to 32 years (Table 2). All SRs included both men and women, and often a generally healthy population [17, 21, 24]. In other SRs, the population was mixed or information on health status was lacking [18–20, 22, 23, 25, 26].

**Table 2:** Characteristics of the included systematic reviews

Author, year	Study type, study period	Study population	Exposition	Outcome	Effect estimates	Heterogeneity estimators	NutriGrade rating	AMSTAR 2 rating
Ma 2024 [23]	–SR with MA of cohort studies –published until 03/2023 –Follow-up: 2.5–24.6 yrs	–Both sexes –“of any health status”, but free of CVD –age range 40–83 yrs		CVD and stroke events	RR random effects model (at least five study comparisons available) or fixed effects models (fewer than five study comparisons available)			Low
	15 cohort studies	n = 286,075 events: 10,278	Total protein	CVD events	0.90 (0.84–0.97)^a^ *P* = 0.006	I^2^ = 38.8% *P* NP	Moderate	
	3 cohort studies	n NP	Total protein: dose–response analysis	CVD events	No significant association *P* = 0.94	NP		
	5 cohort studies	n = 212,582 events: 4,175	Animal protein	CVD events	0.77 (0.63–0.92) *P* = 0.008	I^2^ = 52.2% *P* NP	Moderate	
	5 cohort studies	n = 212,582 events: 4,175	Plant protein	CVD events	0.85 (0.77–0.95) *P* = 0.003	I^2^ = 0,0% *P* NP	Low	
	5 cohort studies	n NP events: 4,899	Total protein	Stroke events	0.84 (0.75–0.95) *P* = 0.004	I^2^ = 0,0% *P* NP	Low	
Lamberg-Allardt 2023 [23]	–SR without MA of cohort studies –published until 05/2022 –follow-up: 74,776 person-yrs							High
	1 cohort study	- no information on included sexes - healthy - mean age 61.6 yrs n = 5,873 events: NP	Animal vs plant protein (replacement of 5 E%)	CHD incidence	HR (95% CI): 0.69 (0.38-1.23)	NA	Moderate	
Mantzouranis 2023 [25]	SR with MA of cohort studies published until 02/2023 follow-up: 4.8–32 yrs	–Both sexes –healthy, with T2D or HTN –mean age 60.4 yrs –mean BMI 26.1 kg/m^2^	Total protein diet: high (> 18 E%) *vs* normal protein intake		OR (95% CI), random effects model			High
	13 cohort studies	n = 525,047 events: 21,906		Total CVD incidence & mortality (Composite outcome: non-fatal myocardial infarction, non-fatal stroke & cardiovascular death)	0.87 (0.70-1.07) P = 0.19	I^2^ = 97 % Chi^2^ = 435.77 Tau^2^ = 0.14 P < 0.00001	Moderate	
	3 cohort studies	n = 90,231 events: 3,436		Stroke incidence	1.02 (0.94–1.10) *P* = 0.66	I^2^ = 0% Chi^2^ = 0.19 Tau^2^ = 0.00 *P* = 0.91	Low	
Zuo 2023 [26]	SR without MA of cohort studies published until 10/2022 follow-up: 2.5 yrs	–no information on included sexes –no information on age or health status –aged > 18 yrs	Total soy protein: highest (> = 11.19 g/d) *vs* lowest (< 4.5 g/d) intake	CHD	RR (95% CI)			Moderate
	1 cohort study	n = 64,915 events: 62			0.25 (0.10, 0.63)		High	
Mousavi 2022 [21]	–SR with MA of cohort studies –published until 04/2020 –follow-up: 4–22 yrs	–Both sexes –general population –aged 30–79 yrs			RR (95% CI), random effects model			High
	6 cohort studies	n = 375,207 events: 9,193	Total protein: highest *vs* lowest intake	CHD incidence	0.97 (0.90, 1.05) *P* NP	I^2^ = 9.1% *P* = 0.36	Low	
	4 cohort studies	n = 212,035 events: 7,270	Total protein: dose–response analysis	CHD incidence & mortality	1.01 (0.96, 1.06) per 5% increase in energy intake from total protein P = 0.58 *P*_nonlinearity_ = 0.69	I^2^ = 0%	Low	
	3 cohort studies	n = 138,828 events: 4,945	Animal protein: highest *vs* lowest intake	CHD incidence	1.09 (0.97, 1.22) *P* NP	I^2^ = 0.0% *P* = 0.69	Low	
	3 cohort studies	n = 138,828 events: 4,945	Plant protein: highest *vs* lowest intake	CHD incidence	1.03 (0.86, 1.24) *P* NP	I^2^ = 39.7% *P* = 0.19	Low	
	2 cohort studies	n = 51,704 events: 3,030	Plant protein: dose–response analysis	CHD incidence & mortality	0.86 (0.64–1.16) per 5% increase in energy intake from plant protein	I^2^ = 0%	Low	
Boushey 2020 [22]	SR without MA of cohort studies –published between 01/2000 and 10/2019 –follow-up: 4–22 yrs	–Both sexes –healthy and/or at risk of chronic disease –aged > = 19 yrs				NA		High
	1 cohort study	n = 7,216	Total protein (14 *vs* 20 En% protein)	Total CVD	↔		Moderate	
	1 cohort study	n = 7,216	Animal protein	Total CVD	↑		High	
	1 cohort study	n = 7,216	Plant protein	Total CVD	↔		Moderate	
	3 cohort studies	n = 138,828	Total protein (12–16 *vs* 23 En% protein)	CHD	↔: 3 studies		Moderate	
	1 cohort study	n = 82,802	Animal protein (16 *vs* 24 En% protein)	CHD	↔		Moderate	
	3 cohort studies	n = 90,231	Total protein (12–14 *vs* 19–23 En% protein)	Stroke	↓: 1 study ↔: 2 studies		Moderate	
	1 cohort study	n = 34,670	Animal protein	Stroke	↓		High	
	1 cohort study	n = 34,670	Plant protein	Stroke	↔		Moderate	
Yan 2017 [20]	–SR with MA of 4 cohort studies – published until 02/2016 –follow-up: 2.5–14.7 yrs	–Both sexes – age NP –n = 260,607 –events: 7,384	Soya protein: highest *vs* lowest intake	Total CVD	RR (95% CI), random effects model 1.08 (0.89, 1.30) *P* NP	I^2^ = 72.6% *P* = 0.006	Low	Moderate
Zhang 2016 [19]	–SR with MA of cohort studies – published until 06/2016 –follow-up: 5–26 yrs	–Both sexes –no information on health status – aged 30–101 yrs	Highest *vs* lowest protein intake	Stroke incidence and mortality	RR (95% CI)^b^			Low
	12 cohort studies	n = 528,982	Total protein		0.98 (0.89, 1.07) *P* NS	I^2^ = 66.5% Chi^2^ = 44.72 *P* = 0.000	Moderate	
	8 cohort studies	n NP	Animal protein		0.94 (0.75, 1.17) *P* NP	I^2^ = 74.1% Chi^2^ = 30.89 *P* = 0.000	Low	
	8 cohort studies	n NP	Plant protein		0.90 (0.82, 0.99) *P* NP	I^2^ = 0.0% Chi^2^ = 2.11 *P* = 0.977	Low	
Zhang 2014 [18]	–SR with MA of cohort studies – published until 11/2013 –follow-up: 10.4 18 yrs	–Both sexes –no information on health status –aged 34–89 yrs	Highest *vs* lowest protein intake	Stroke incidence and mortality	RR (95% CI), fixed effects model or random effects model in the presence of heterogeneity			Moderate
	7 cohort studies	n = 254,489	Total protein: high *vs* low analysis		0.80 (0.66, 0.99) *P* NP	I^2^ = 61.1% Chi^2^ = 15.44 *P* = 0.017	Moderate	
	5 cohort studies	n = 129,799	Total protein: dose–response analysis		0.74 (0.65, 0.84) per 20-g/d increment *P* NP *P*_nonlinearity_ = 0.93			
	5 cohort studies	n = 172,900	Animal protein		0.71 (0.50, 0.99) *P* NP	I^2^ = 69.4% Chi^2^ = 13.07 *P* = 0.011	Moderate	
	5 cohort studies	n = 172,900	Plant protein		0.88 (0.76, 1.02) *P* NP	I^2^ = 0.0% Chi^2^ = 1.17 *P* = 0.884	Low	
Pedersen 2017 [17]	–SR without MA of cohort studies –published between 01/2000–12/2011 –follow-up: 18 mo to 20 yrs	–Both sexes –generally healthy –aged 34–79 yrs	Higher *vs* lower protein intake			NA		Moderate
	3 cohort studies	n = 207,132 events: 8,870	Total protein	CHD incidence and mortality	2 out of 3 studies: no association *“The evidence is assessed as inconclusive regarding the relationship between protein intake and risk of coronary heart disease”*		Moderate	
	2 cohort studies	n = 126,762 events: 4,953	Animal protein, plant protein		2 out of 2 studies: no association		Moderate^c^	
	2 cohort studies	n = 129,724	Total protein, animal protein, plant protein	Stroke incidence and mortality	2 out of 2 studies: no association *“The evidence is assessed as inconclusive regarding the relation of protein intake to risk of stroke”*		Moderate^d^	

AMSTAR 2, A measurement tool to assess systematic reviews; CHD, coronary heart disease; CI, confidence interval; d, day(s); En%, energy percentage; MA, meta-analysis; mo, month; NA, not applicable; NP, not provided; NS, not statistically significant; RR, relative risk; SMD, standardised mean difference; SR, systematic review; wk, week; WMD, weighted mean difference; yr, year

### Methodological quality

Overall scores of AMSTAR 2 for each included SR are summarised in Table 2. Supplementary Material 5 provides a more detailed overview showing the assessments of each individual item. The methodological quality of the included SRs as assessed by AMSTAR 2 was high for four SRs [21, 22, 24, 25], moderate for four SRs [17, 18, 20, 26], and low for two SRs [19, 23].

### Associations/effects of protein intake; and outcome-specific certainty of the evidence

There were ten SRs of cohort studies on the association of protein intake and CVD [17–26]. The study characteristics of the included SRs are shown in Table 2. Outcome-specific certainty of evidence was low for 10, and moderate for 19 of the associations; in three cases it was ranked as high, and none was rated as very low. Overall scores obtained from NutriGrade rating for each SR are summarised in Table 2. Supplementary Material 6 provides more details and shows the assessments of each individual item.

### Associations between the intake of total, animal, and plant protein and the risk of CHD

#### Total protein intake

The association between total protein intake and the risk of CHD was investigated in three SRs, among them one with MA [21] and two without MA [17, 22] (Table 2). The SR with MA by Mousavi et al. [21], which included 6 prospective cohort studies, found no significant difference in the risk of CHD between the highest and lowest category of protein intake (RR = 0.97, 95% CI = 0.90–1.05). In the SR without MA by Boushey et al. [22], none of the three cohort studies observed an association between total protein intake and the risk of CHD. This is in line with two out of three cohort studies included by Pedersen et al. [17]. Moreover, according to the dose–response-analysis of Mousavi et al. [21], a 5% increase in energy intake from total protein was not associated with a change in CHD risk.

To summarise, all SRs did not find an association between total protein intake and the risk of CHD. Since most SRs reached a moderate outcome-specific certainty of evidence and a high methodological quality, the overall certainty of evidence was graded as “probable” for the absence of an association between total protein intake and the risk of CHD.

#### Animal protein intake

The same SRs analysed the association between the intake of animal protein and the risk of CHD [17, 21, 22]. Again, the MA by Mousavi et al. [21] did not reveal a difference between the highest and lowest category of protein intake from animal origin on the basis of three cohort studies with a total of 138,828 participants. The SRs without MA by Boushey et al. [22] and Pedersen et al. [17] considered only one and two cohort studies, respectively, and none of the cohort studies found an association between the amount of animal protein intake and the risk of CHD.

In summary, none of the SRs found an association between animal protein intake and the risk of CHD. Taking into account that most SRs reached a moderate outcome-specific certainty of evidence and a high methodological quality, the overall certainty of evidence was graded as “probable” for a lack of association between the intake of animal protein and the risk of CHD.

#### Plant protein intake

Three SRs investigated the association between the intake of plant protein and the risk of CHD, one SR with MA [21] and two SRs without [17, 26]. The MA by Mousavi et al. [21] was based on three cohort studies and did not find an association. These findings align with the results from the two cohort studies considered in the SR without MA by Pedersen et al. [17]. Furthermore, the dose–response-analysis of Mousavi et al. [21] did not observe that a 5% daily increase in energy intake from plant protein was associated with changes in CHD risk. However, Zuo et al. [26] observed a significant lower CHD risk under soya protein consumption.

Altogether, the results of most SRs consistently report that there is no association between the intake of plant protein and the risk of CHD. Since most SRs reached a moderate outcome-specific certainty of evidence and all SRs reached at least a moderate methodological quality, the overall certainty of evidence of having no association between plant protein intake and the risk of CHD is rated as “probable”.

#### Plant protein intake versus animal protein

Only one SR without MA investigated animal vs. plant protein and the incidence of CHD. Higher plant protein intake was not associated with a lower risk of CHD when consumed at the expense of animal protein [24]. Due to the limited data base, an evaluation of the overall certainty of evidence did not seem appropriate.

### Associations between the intake of total, animal, and plant protein and the risk of stroke

#### Total protein intake

Six SRs investigated the association between total protein intake and the risk of stroke, among them four SRs with MA [18, 19, 23, 25] and two without MA [17, 22]. Zhang et al. [18] found a 20% lower risk of stroke for the highest vs. the lowest category of protein intake on the basis of seven cohort studies with 254,489 participants and a follow-up between 10.4 and 18 years. This association was dose-dependent; a 20 g/d increment in dietary protein was associated with a 26% lower risk for stroke. Ma et al. [23] found 16% reduced risk for stroke based on 5 cohort studies. In contrast, the other MA that included 12 cohort studies with 528,982 subjects did not find an association, but the follow-up ranged from 5 to 26 years [19], thus being much broader (follow-up: 10.4–18 years) than in the MA of Zhang et al. 2014 [18]. The differentiation between ischaemic and haemorrhagic stroke as conducted by Zhang et al. [19] did not reveal an association for a stroke subtype. The recently published MA by Mantzouranis et al. [25] also did not find an association between total protein intake and the risk of stroke based on three cohort studies. The SR without MA by Boushey et al. [22] also considered three cohort studies; one of them found a decreased risk of stroke by increasing the amount of total protein, and two did not reveal an association. Both cohort studies considered by Pedersen et al. [17] did also find no association.

To summarise, most SRs did not find an association between total protein intake and the risk of stroke. The majority of SRs had a moderate outcome-specific certainty of evidence and the majority reached at least a moderate methodological quality. Therefore, the overall certainty of evidence was rated as “possible” that no association exists between total protein intake and the risk of stroke.

#### Animal protein intake

Four SRs investigated the association between animal protein intake and the risk of stroke. One SR with MA including five cohort studies observed an inverse association between animal protein intake and the risk of stroke [18]. The SR without MA of Boushey et al. [22] also found an inverse association, which, however, was based on a single cohort study. The other SRs with MA [19] and without MA [17] did not reveal an association.

In total, two of four of the SRs found a lower risk of stroke with higher animal protein intake, whereas the remaining two SRs did not. Therefore, the overall certainty of evidence was graded as “insufficient”.

#### Plant protein intake

Four SRs investigated the association between the intake of plant protein and the risk of stroke; two SRs were conducted with MA [18, 19] and two without MA [17, 22]. The SR of Zhang et al. [19] which was based on eight cohort studies found an inverse association between plant protein intake and the risk of stroke. Conversely, an earlier MA conducted by a different research group [18] (based on five cohort studies) did not report an association. Similarly, no association was identified in any of the primary studies included in both SRs without MA [17, 22].

To summarise, three of four SRs did not observe an association between the intake of plant protein and the risk of stroke. As most SRs had at least a low outcome-specific certainty of evidence and at least a moderate methodological quality, the overall certainty of evidence was graded as “possible” that no association exists between the intake of plant protein and the risk of stroke.

### Associations between the intake of total, animal, and plant protein and the risk of total CVD

#### Total protein intake

The association between total protein intake and the risk of CVD was investigated in three SRs, among them two with MA [23, 25] and one without MA [22]. Only Ma et al. [23] found that a high total protein intake was associated with a 12% lower risk for CVD based on 15 cohort studies. In contrast, Mantzouranis et al. [25] did not observe an association between total protein intake and CVD based on 13 cohort studies. However, their CVD endpoint also included CVD mortality. The SR without MA of Boushey et al. [22] considered one cohort study with 7,216 participants. Comparing the highest (20% of total energy) vs. lowest total protein intake (14%) category, no difference in total CVD risk was observed.

To summarise, two of three SRs did not find an association between total protein intake and total CVD. All SRs had a moderate outcome-specific certainty of evidence and the majority reached at least a moderate methodological quality. Therefore, the overall certainty of evidence was rated as “possible” that no association exists between total protein intake and total CVD risk.

#### Animal protein intake

The association between animal protein intake and the risk of CVD was investigated in one SR with MA [23] and in one SR without MA [22]. Ma et al. [23] found that a high animal protein intake was associated with a 23% lower risk for CVD including 5 cohort studies. Based on a single cohort study of 7,216 participants, Boushey et al. [22] found that animal protein intake was associated with a higher risk of CVD.

Overall, one of the two SRs found a lower risk of CVD with higher consumption of animal protein, while the opposite was observed in the other SR. Therefore, the overall certainty of evidence was graded as “insufficient”.

#### Plant protein intake

The association between plant protein intake and the risk of total CVD was investigated in three SRs, two with MA [20, 23] and one without [22]. The MA by Yan et al. [20] was based on four cohort studies with a total of 260,607 participants; it was restricted to soya protein. No association was observed comparing the highest vs. lowest intake category. The recently published MA of Ma et al. [23] found that a high plant protein intake was associated with a 15% lower risk for CVD based on 5 cohort studies. The SR without MA by Boushey et al. [22] considered a single cohort study with 7,216 participants; the intake of plant protein was not associated with the risk of CVD.

To summarise, two of three SRs did not find an association between plant protein intake and total CVD. The majority of SRs had at least a low outcome-specific certainty of evidence and the majority reached at least a moderate methodological quality. Therefore, the overall certainty of evidence was rated as “possible” that no association exists between plant protein intake and total CVD risk.

## Discussion

This umbrella review evaluated the evidence on the impact of total dietary protein intake as well as of animal and plant protein intake on CHD, stroke and total CVD in the adult population. Based on 10 SRs of prospective cohort studies we found no evidence for an association between protein intake and the risk of CHD, stroke and total CVD, respectively. As the methodological quality of the included SRs was often rated at least as “moderate”, the overall certainty of evidence for the lack of an association between the intake of total protein, animal protein or plant protein and the risk of CHD was judged as “probable”. In the case of stroke and total CVD, the overall certainty of evidence for the absence of an association between total protein or plant protein and the risk of stroke or total CVD was rated as “possible”. For stroke and animal protein as well as total CVD and animal protein, the overall certainty of evidence was rated as insufficient due to the limited availability of data.

In contrast to our results, there is evidence that the risk of CVD outcomes might be affected by different dietary protein sources (such as dairy, legumes, meat) which provide not only protein but also a complex mixture of ingredients. It can be assumed that regarding multifactorial diseases such as CVD, the effects of a mixture of synergistic substances may be larger than that of a single nutrient such as protein. For example, an increase in red and processed meat, the main sources of animal protein, was associated with an increased risk of CVD [31], which may reflect the effect of for example sodium and SAFA from animal protein sources on the risk of CVD outcomes. On the other hand, regular intake of fatty fish, another source of animal protein, is associated with a decreased risk of CVD which can be explained by the protective effects of long-chain omega-3 fatty acids [32]. Similarly, the consumption of whole grains, legumes, and nuts, major sources of plant protein, was associated with a lower risk of CVD [33, 34], which may be explained by health-promoting ingredients such as dietary fibre, unsaturated fatty acids, vitamins, potassium, magnesium, and a low sodium content. This is also underlined by the results of Yan et al. [20] who investigated the association between the consumption of soya foods, as well as soya protein, and the risk of CVD. The consumption of soya foods was associated with a reduced risk of CVD, but no association was found for soya protein itself. Many health-promoting compounds in soya foods might account for these inverse associations, such as dietary fibre, unsaturated fatty acids, and isoflavones. Taken together, it remains unclear whether protein itself has an influence on the CVD endpoints or whether other ingredients in protein-rich foods either mask or modulate possible effects of dietary protein.

From the mechanistic point of view, there is accumulating evidence that specific amino acids and their metabolites have been implicated in various processes hallmarking atherosclerotic CVD. In particular, the importance of arginine and its metabolites, homoarginine and polyamines, branched-chain amino acids, glycine and aromatic amino acids, is discussed due to their implication in impaired lipid metabolism, endothelial dysfunction, increased inflammatory response and development of a necrotic core [35].

Hypertension is a main risk factor for CHD and especially for stroke. Dietary measures to reduce elevated blood pressure reduce the risk of both conditions [36]. A recent umbrella review of our guideline group of Boeing et al. [37] evaluating the role of dietary intake of total, animal and plant protein on blood pressure and hypertension, based on prospective cohort studies and RCTs, revealed “possible” evidence for no link between total, animal, and plant protein intake and blood pressure. This may contribute to the lack of association between protein intake and CHD and/or stroke, as observed in the present umbrella review.

A further risk factor for CVD is overweight/obesity, especially visceral obesity. It has been suggested that an increased dietary protein intake might decrease the risk of obesity, for example, by increasing diet-induced thermogenesis and by increasing satiety [38]. However, another umbrella review of our guideline group evaluating the impact of protein intake (amount and type) on body weight based on prospective cohort studies and predominately RCTs found that there is “possible” evidence that in studies without energy restriction the amount of protein intake does not affect body weight in the general adult population [39]. Therefore, an influence of protein intake on the intermediate risk factor obesity seems unlikely.

Our umbrella review has some limitations. First, the evaluation of evidence was solely based on SRs of prospective cohort studies since SRs of RCTs were not available. This type of study cannot prove a causal relationship between protein intake and CVD. In addition, data on food intake are based on self-reporting by the study participants (e.g., food frequency questionnaires, 24-h recalls), meaning that recall bias, under- or overreporting, or incorrect estimation of portion sizes cannot be ruled out. Biomarkers as an objective measurement for protein intake would be desirable in this context, but were not used in the studies considered. On the other hand, it is unlikely that RCTs on the effects of protein on CVD endpoints will be available in the future, particularly because these studies would require a very long intervention period and a large number of participants. In addition, the feasibility and practicability of such controlled nutritional interventions over such a long period of time would not be given.

Second, a distinction between dietary proteins was only made between protein of animal and of plant origin. As already highlighted, different protein sources of animal origin (e.g., meat, milk, dairy, and fatty fish) may differently modify CVD endpoints, so that possible effects can be mutually compensating each other in an omnivorous diet setting considered in the included studies. In addition to the present umbrella review on protein intake and CVD endpoints, umbrella reviews on the association between the intake of certain food groups and the incidence of CVD are needed. This is particularly relevant for the scientific derivation of evidence-based food-based dietary guidelines.

We included the results of all relevant SRs, regardless of overlap as our purpose was to present and describe the current body of SR evidence. Having assessed the extent of primary study overlap between the SRs, bias due to multiple inclusion of the same primary studies in different SRs is unlikely, as the primary study overlap is only small with 9%.

Our umbrella consists of 10 SRs, considering in total 34 primary studies. We identified two recent primary studies investigating the association between plant- and animal-derived protein intake and incident CVD. Both studies found no significant associations between protein consumption and CVD outcomes [40, 41].

In conclusion, this umbrella review evaluated the available evidence for the role of dietary protein on CHD, stroke and total CVD in the adult population, yielding mostly “possible” or “probable” evidence for no association between total protein, animal protein and plant protein intake and the three endpoints.

## Supplementary Information

Below is the link to the electronic supplementary material.

Supplementary file1 (XLSX 15 kb)

Supplementary file2 (XLSX 16 kb)

Supplementary file3 (XLSX 14 kb)

Supplementary file4 (DOCX 41 kb)

Supplementary file5 (DOCX 42 kb)

Supplementary file6 (DOCX 31 kb)

Supplementary Material 6

Supplementary Material 7

Supplementary Material 8

## Abbreviations

AMSTAR 2: A measurement tool to assess systematic reviews 2
CI: Confidence interval
CHD: Coronary heart disease
CVD: Cardiovascular diseases
MA: Meta-analysis/meta-analyses
RCT: Randomised controlled trial
SAFA: Saturated fatty acids
SR: Systematic review
