# Supplementary material for: Protein intake and cardiovascular diseases: an umbrella review of systematic reviews for the evidence-based guideline on protein intake of the German Nutrition Society
Source: Eur J Nutr. 2025 Aug 13;64(6):254. doi: 10.1007/s00394-025-03746-2 (PMC12350521; doi:10.1007/s00394-025-03746-2)
Supplement: Supplementary file 8 — Supplementary Material 7 [file 394_2025_3746_MOESM8_ESM.docx]

Supplementary Material 7. List of excluded articles with rationale for exclusion.

| **Reference** | **Rationale for exclusion** |
| --- | --- |
| Adegbola A, Behrendt C-A, Zyriax B-C et al. (2022) The impact of nutrition on the development and progression of peripheral artery disease: a systematic review. Clin Nutr 41:49–70. | Irrelevant study type |
| Ajala O, English P, Pinkney J (2013) Systematic review and meta-analysis of different dietary approaches to the management of type 2 diabetes. Am J Clin Nutr 97:505–516. | Irrelevant outcome |
| Al-Shaar L, Wang M, Willett W et al. (2021) Red and processed meat and alternative protein sources in relation to risk of fatal coronary heart disease: a pooled analysis of 16 prospective cohort studies. Circulation 143, Suppl 1. | Only abstract |
| Alexander DD, Bylsma LC, Vargas AJ et al. (2016) Dairy consumption and CVD: a systematic review and meta-analysis. Br J Nutr 115:737–750. | Irrelevant exposure |
| Alexander DD, Miller PE, Vargas AJ et al. (2016) Meta-analysis of egg consumption and risk of coronary heart disease and stroke. J Am Coll Nutr 35:1–13. | Irrelevant exposure |
| Ali Redha A, Valizadenia H, Siddiqui SA et al. (2022) A state-of-art review on camel milk proteins as an emerging source of bioactive peptides with diverse nutraceutical properties. Food Chem 373:131444. | Irrelevant exposure |
| Aljefree N, Ahmed F (2015) Association between dietary pattern and risk of cardiovascular disease among adults in the Middle East and North Africa region: a systematic review. Food Nutr Res 59:27486. | Irrelevant exposure |
| Arnesen EK, Thorisdottir B, Bärebring L et al. (2023) Nuts and seeds consumption and risk of cardiovascular disease, type 2 diabetes and their risk factors: a systematic review and meta-analysis. Food Nutr Res 67:8961. | Relevant diet-disease relationship not investigated |
| Astrup A, Geiker NRW, Magkos F (2019) Effects of full-fat and fermented dairy products on cardiometabolic disease: food is more than the sum of its parts. Adv Nutr 10:924S–930S. | Irrelevant study type |
| Attaye I, Warmbrunn MV, Boot ANAF et al. (2022) A systematic review and meta-analysis of dietary interventions modulating gut microbiota and cardiometabolic diseases-striving for new standards in microbiome studies. Gastroenterology 162:1911–1932. | Relevant diet-disease relationship not investigated |
| Barrett EM, Batterham MJ, Ray S et al. (2019) Whole grain, bran and cereal fibre consumption and CVD: a systematic review. Br J Nutr 121:914–937. | Irrelevant exposure |
| Battaglia Richi E, Baumer B, Conrad B et al. (2015) Health risks associated with meat consumption: a review of epidemiological studies. Int J Vitam Nutr Res 85:70–78. | Irrelevant study type |
| Benkhedda K, Boudrault C, Sinclair S et al. (2015) A systematic review and meta‐analysis of the effects of soy products on blood cholesterol levels. FASEB J 29:923.14. | Only abstract |
| Bhupathi V, Mazariegos M, Cruz Rodriguez JB et al. (2020) Dairy intake and risk of cardiovascular disease. Curr Cardiol Rep 22:11. | Irrelevant study type |
| Blanco Mejia S, Messina M, Li SS et al. (2019) A meta-analysis of 46 studies identified by the FDA demonstrates that soy protein decreases circulating LDL and total cholesterol concentrations in adults. J Nutr 149:968–981. | Irrelevant outcome |
| Bloomfield HE, Kane R, Koeller E et al. (2015) Benefits and harms of the Mediterranean diet compared to other diets. Department of Veterans Affairs (US), Washington (DC). | Irrelevant exposure |
| Bouaziz W, Schmitt E, Kaltenbach G et al. (2015) Health benefits of endurance training alone or combined with diet for obese patients over 60: a review. Int J Clin Pract 69:1032–1049. | Irrelevant exposure |
| Bryant L, Rangan A, Grafenauer S (2022) Lupins and health outcomes: a systematic literature review. Nutrients 14:327. | Irrelevant outcome |
| Calabrese I, Riccardi G (2019) Effectiveness of changes in diet composition on reducing the incidence of cardiovascular disease. Curr Cardiol Rep 21:88. | Irrelevant exposure |
| Camargo LdR, Doneda D, Oliveira VR (2020) Whey protein ingestion in elderly diet and the association with physical, performance and clinical outcomes. Exp Gerontol 137:110936. | Irrelevant outcome |
| Caterina R de, Salvatore T, Marchioli R (2016) All cholesterol-lowering interventions are expected to reduce stroke: confirmatory data from IMPROVE-IT. Data Brief 7:1541–1550. | Irrelevant study type |
| Chalvon-Demersay T, Azzout-Marniche D, Arfsten J et al. (2017) A systematic review of the effects of plant compared with animal protein sources on features of metabolic syndrome. J Nutr 147:281–292. | Irrelevant outcome |
| Chen G-C, Wang Y, Tong X et al. (2017) Cheese consumption and risk of cardiovascular disease: a meta-analysis of prospective studies. Eur J Nutr 56:2565–2575. | Irrelevant exposure |
| Chen W, Zhang S, Hu X et al. (2023) A review of healthy dietary choices for cardiovascular disease: from individual nutrients and foods to dietary patterns. Nutrients 15:4898. | Irrelevant study type |
| Chen Z, Ahmed M, Ha V et al. (2021) Dairy product consumption and cardiovascular health: a systematic review and meta-analysis of prospective cohort studies. Adv Nutr. 13:439–454. | Irrelevant exposure |
| Chen Z, Glisic M, Song M et al. (2020) Dietary protein intake and all-cause and cause-specific mortality: results from the Rotterdam Study and a meta-analysis of prospective cohort studies. Eur J Epidemiol 35:411–429. | Irrelevant outcome |
| Chiavaroli L, Nishi SK, Khan TA et al. (2018) Portfolio dietary pattern and cardiovascular disease: a systematic review and meta-analysis of controlled trials. Prog Cardiovasc Dis 61:43–53. | Irrelevant exposure |
| Chrysant SG, Chrysant GS (2022) Inverse association of poultry, fish, and plant protein consumption with the incidence of cardiovascular disease. Cardiol Rev 30:247–252. | Irrelevant outcome |
| Companys J, Pla-Pagà L, Calderón-Pérez L et al. (2020) Fermented dairy products, probiotic supplementation, and cardiometabolic diseases: a systematic review and meta-analysis. Adv Nutr 11:834–863. | Relevant diet-disease relationship not investigated |
| Connolly G, Clark CM, Campbell RE et al. (2022) Poultry consumption and human health: how much is really known? A systematically searched scoping review and research perspective. Adv Nutr 13:2115–2124. | Irrelevant exposure |
| Corella D, Ordovas JM (2012) Dairy consumption, plasma lipoproteins, and cardiovascular risk: finding the balance. Curr Cardiovasc Risk Rep 6:35–44. | Irrelevant study type |
| Dahl IK, Dalgård C (2021) Sami dietary habits and the risk of cardiometabolic disease: a systematic review. Int J Circumpolar Health 80:1873621. | Irrelevant exposure |
| De Goede J, Geleijnse JM, Pan A et al. (2016) Dairy consumption and risk of stroke: a systematic review and dose-response meta-analysis of prospective cohort studies. Circulation 133, Suppl 1:320. | Only abstract |
| de Morais Cardoso L, Pinheiro SS, Martino HSD et al. (2017) Sorghum (Sorghum bicolor L.): nutrients, bioactive compounds, and potential impact on human health. Crit Rev Food Sci Nutr 57:372–390. | Relevant diet-disease relationship not investigated |
| Deng C, Lu Q, Gong B et al. (2018) Stroke and food groups: an overview of systematic reviews and meta-analyses. Public Health Nutr 21:766–776. | Irrelevant exposure |
| Deupmann A-K, Debus ES, Grundmann RT (2021) Evidenzbasierte Ernährung bei zerebrovaskulären und kardiovaskulären Erkrankungen. Teil 2 Zerebrovaskuläre Erkrankungen. Gefässchirurgie 26:392–401. | Irrelevant study type |
| Doundoulakis I, Farmakis I, Christoglou M et al. (2021) Effects of dietary interventions on cardiovascular outcomes: a network meta-analysis. Clin Nutr ESPEN 46:S739. | Only abstract |
| Dreher ML (2021) A comprehensive review of almond clinical trials on weight measures, metabolic health biomarkers and outcomes, and the gut microbiota. Nutrients 13:1968. | Irrelevant study type |
| Drouin-Chartier J-P, Brassard D, Tessier-Grenier M et al. (2016) Systematic review of the association between dairy product consumption and risk of cardiovascular-related clinical outcomes. Adv Nutr 7:1026–1040. | Irrelevant exposure |
| Dugani SB, Hydoub YM, Ayala AP et al. (2021) Risk Factors for premature myocardial infarction: a systematic review and meta-analysis of 77 studies. Mayo Clin Proc Innov Qual Outcomes 5:783–794. | Irrelevant exposure |
| Ekmekcioglu C, Wallner P, Kundi M et al. (2018) Red meat, diseases, and healthy alternatives: a critical review. Crit Rev Food Sci Nutr 58:247–261. | Relevant diet-disease relationship not investigated |
| Eslami O, Zarei M, Shidfar F (2020) The association of dietary patterns and cardiorespiratory fitness: a systematic review. Nutr Metab Cardiovasc Dis 30:1442–1451. | Irrelevant exposure |
| Esmaeili Nadimi A, Ahmadi Z, Falahati-Pour SK et al. (2020) Physicochemical properties and health benefits of pistachio nuts. Int J Vitam Nutr Res 90:564–574. | Relevant diet-disease relationship not investigated |
| Fadnes LT, Balakrishna R (2024) Nuts and seeds - a scoping review for Nordic Nutrition Recommendations 2023. Food Nutr Res 68:10483. | Relevant diet-disease relationship not investigated |
| Ferreira H, Vasconcelos M, Gil AM et al. (2021) Benefits of pulse consumption on metabolism and health: a systematic review of randomized controlled trials. Crit Rev Food Sci Nutr 61:1–12. | Irrelevant outcome |
| Fontecha J, Visitación Calvo M, Juarez M et al. (2019) Milk and dairy product consumption and cardiovascular diseases: an overview of systematic reviews and meta-analyses. Adv Nutr 10, Suppl 2:S164–S189. | Irrelevant study type |
| Francini-Pesenti F. (2018) Nutrition in the healthy population. Acta Myologica 37:39. | Only abstract |
| Ge L, Sadeghirad B, Ball GDC et al. (2020) Comparison of dietary macronutrient patterns of 14 popular named dietary programmes for weight and cardiovascular risk factor reduction in adults: systematic review and network meta-analysis of randomised trials. BMJ 369:m696. | Irrelevant outcome |
| Gholami F, Khoramdad M, Esmailnasab N et al. (2017) The effect of dairy consumption on the prevention of cardiovascular diseases: a meta-analysis of prospective studies. J Cardiovasc Thorac Res 9:1–11. | Irrelevant exposure |
| Gholami F, Khoramdad M, Shakiba E et al. (2017) Subgroup dairy products consumption on the risk of stroke and CHD: a systematic review and meta-analysis. Med J Islam Repub Iran 31:25. | Irrelevant exposure |
| Gille D, Schmid A, Walther B et al. (2018) Fermented food and non-communicable chronic diseases: a review. Nutrients 10:448. | Irrelevant study type |
| Giosuè A, Calabrese I, Vitale M et al. (2022) Consumption of dairy foods and cardiovascular disease: a systematic review. Nutrients 14:831. | Irrelevant exposure |
| Giosuè A, Recanati F, Calabrese I et al. (2022) Good for the heart, good for the Earth: proposal of a dietary pattern able to optimize cardiovascular disease prevention and mitigate climate change. Nutr Metab Cardiovasc Dis 32:2772–2781. | Irrelevant exposure |
| Givens DI (2018) Review: dairy foods, red meat and processed meat in the diet: implications for health at key life stages. Animal 12:1709–1721. | Irrelevant study type |
| Godos J, Tieri M, Ghelfi F et al. (2020) Dairy foods and health: an umbrella review of observational studies. Int J Food Sci Nutr 71:138–151. | Irrelevant study type |
| Goede J de, Soedamah-Muthu SS, Pan A et al. (2016) Dairy consumption and risk of stroke: a systematic review and updated dose-response meta-analysis of prospective cohort studies. J Am Heart Assoc 5:e002787. | Irrelevant exposure |
| Guasch-Ferré M, Satija A, Blondin SA et al. (2019) Meta-analysis of randomized controlled trials of red meat consumption in comparison with various comparison diets on cardiovascular risk factors. Circulation 139:1828–1845. | Irrelevant outcome |
| Gupta M, Asfaha DM, Ponnaiah G (2023) Millets: a nutritional powerhouse with anti-cancer potential. Cureus 15:e47769. | Irrelevant study type |
| Hajizadeh-Sharafabad F, Sharifi Zahabi E, Tarighat-Esfanjani A (2022) Role of whey protein in vascular function: a systematic review and meta-analysis of human intervention studies. Br J Nutr 128:1–14. | Irrelevant outcome |
| He S, Stein AD (2021) Early-Life nutrition interventions and associated long-term cardiometabolic outcomes: a systematic review and meta-analysis of randomized controlled trials. Adv Nutr 12:461–489. | Irrelevant population |
| Hidayat K, Chen J-S, Wang H-P et al. (2022) Is replacing red meat with other protein sources associated with lower risks of coronary heart disease and all-cause mortality? A meta-analysis of prospective studies. Nutr Rev 80:1959–1973. | Irrelevant exposure |
| Hidayat K, Yu L-G, Yang J-R et al. (2020) The association between milk consumption and the metabolic syndrome: a cross-sectional study of the residents of Suzhou, China and a meta-analysis. Br J Nutr 123:1013–1023. | Irrelevant exposure |
| Hong JY, Kim MK, Yang N (2024) Mushroom consumption and cardiometabolic health outcomes in the general population: a systematic review. Nutr Res Pract 18:165–179. | Relevant diet-disease relationship not investigated |
| Hooper L, Martin N, Jimoh OF et al. (2020) Reduction in saturated fat intake for cardiovascular disease. Cochrane Database Syst Rev 5:CD011737. | Irrelevant exposure |
| Hu D, Huang J, Wang Y et al. (2014) Dairy foods and risk of stroke: a meta-analysis of prospective cohort studies. Nutr Metab Cardiovasc Dis 24:460–469. | Irrelevant exposure |
| Iacoviello L, Bonaccio M, Cairella G et al. (2018) Diet and primary prevention of stroke: systematic review and dietary recommendations by the ad hoc Working Group of the Italian Society of Human Nutrition. Nutr Metab Cardiovasc Dis 28:309–334. | Irrelevant study type |
| Jakobsen MU, Trolle E, Outzen M et al. (2021) Intake of dairy products and associations with major atherosclerotic cardiovascular diseases: a systematic review and meta-analysis of cohort studies. Sci Rep 11:1303. | Irrelevant exposure |
| Jayachandran M, Xu B (2019) An insight into the health benefits of fermented soy products. Food Chem 271:362–371. | Irrelevant study type |
| Jellinger PS, Handelsman Y, Rosenblit PD et al. (2017) American Association of Clinical Endocrinologists and American College of Endocrinology: guidelines for management of dyslipidemia and prevention of cardiovascular disease. Endocr Pract 23, Suppl 2:1–87. | Irrelevant outcome |
| Jellinger PS, Handelsman Y, Rosenblit PD et al. (2017) American Association of Clinical Endocrinologists and American College of Endocrinology guidelines for management of dyslipidemia and prevention of cardiovascular disease - executive summary. Endocr Pract 23:479–497. | Irrelevant outcome |
| Kalyoncu ZB, Pars H, Bora-Güneş N et al. (2014) A systematic review of nutrition-based practices in prevention of hypertension among healthy youth. Turk J Pediatr 56:335–346. | Irrelevant outcome |
| Kaur A, Kehinde BA, Sharma P et al. (2021) Recently isolated food-derived antihypertensive hydrolysates and peptides: a review. Food Chem 346:128719. | Irrelevant study type |
| Kerley CP (2019) Dietary patterns and components to prevent and treat heart failure: a comprehensive review of human studies. Nutr Res Rev 32:1–27. | Relevant diet-disease relationship not investigated |
| Kern HJ, Mitmesser SH (2017) Role of nutrients in metabolic health: updates in 2016. FASEB J 31:789.10–789.10. | Only abstract |
| Key J, Cantarero A, Cohen D et al. (2016) The dairy fat paradox: a systematic review of the evidence. Top Clin Nutr 31:280–295. | Irrelevant exposure |
| Kim K, Hyeon J, Lee SA et al. (2017) Total, red, processed, and white meat intake and stroke incidence and mortality: a systematic review and meta-analysis of cohort studies. Eur Stroke J 2, Suppl 1:445. | Irrelevant exposure |
| Kirkpatrick CF, Bolick JP, Kris-Etherton PM et al. (2019) Review of current evidence and clinical recommendations on the effects of low-carbohydrate and very-low-carbohydrate (including ketogenic) diets for the management of body weight and other cardiometabolic risk factors: a scientific statement from the National Lipid Association Nutrition and Lifestyle Task Force. J Clin Lipidol 13:689–711.e1. | Irrelevant study type |
| Holven K, Sonestedt E (2024) Milk and dairy products – a scoping review for Nordic Nutrition Recommendations 2023. Food Nutr Res 68:10486. | Relevant diet-disease relationship not investigated |
| Klonizakis M, Bugg A, Hunt B et al. (2021) Assessing the physiological effects of traditional regional diets targeting the prevention of cardiovascular disease: a systematic review of randomized controlled trials implementing Mediterranean, New Nordic, Japanese, Atlantic, Persian and Mexican dietary interventions. Nutrients 13:3034. | Irrelevant exposure |
| Krittanawong C, Isath A, Hahn J et al. (2021) Mushroom consumption and cardiovascular health: a systematic review. Am J Med 134:637–642.e2. | Relevant diet-disease relationship not investigated |
| Lane MM, Davis JA, Beattie S et al. (2021) Ultraprocessed food and chronic noncommunicable diseases: a systematic review and meta-analysis of 43 observational studies. Obes Rev 22:e13146. | Irrelevant exposure |
| Léonil J (2014) Milk bioactive peptides: their interest for cardiovascular diseases and metabolic syndrome prevention. Médecine des Maladies Métaboliques 8:495–499. | Irrelevant language |
| Li Y, Zhou C, Pei H et al. (2013) Fish consumption and incidence of heart failure: a meta-analysis of prospective cohort studies. Chin Med J 126:942–948. | Relevant diet-disease relationship not investigated |
| Lockyer S, La Hunty AE de, Steenson S et al. (2022) Walnut consumption and health outcomes with public health relevance-a systematic review of cohort studies and randomized controlled trials published from 2017 to present. Nutr Rev 81:26–54. | Irrelevant exposure |
| Lonnie M, Laurie I, Myers M et al. (2020) Exploring health-promoting attributes of plant proteins as a functional ingredient for the food sector: a systematic review of human interventional studies. Nutrients 12:2291. | Irrelevant outcome |
| López-Jaramillo P, Otero J, Camacho PA et al. (2018) Reevaluating nutrition as a risk factor for cardio-metabolic diseases. Colomb Med (Cali) 49:175–181. | Irrelevant study type |
| Louisa M, Patintingan CGH, Wardhani BWK (2022) Moringa oleifera Lam. in cardiometabolic disorders: a systematic review of recent studies and possible mechanism of actions. Front Pharmacol 13:792794. | Irrelevant exposure |
| Lovegrove JA, Hobbs DA (2016) New perspectives on dairy and cardiovascular health. Proc Nutr Soc 75:247–258. | Irrelevant study type |
| Macedo RCO, Santos HO, Tinsley GM et al. (2020) Low-carbohydrate diets: effects on metabolism and exercise - a comprehensive literature review. Clin Nutr ESPEN 40:17–26. | Irrelevant exposure |
| Machado de Souza RG, Machado Schincaglia R, Pimentel GD et al. (2017) Nuts and human health outcomes: a systematic review. Nutrients 9:1311. | Irrelevant exposure |
| Mann KD, Pearce MS, Seal CJ (2017) Providing evidence to support the development of whole grain dietary recommendations in the United Kingdom. Proc Nutr Soc 76:369–377. | Irrelevant study type |
| Manolis AS, Manolis TA, Manolis AA et al. (2022) Diet and sudden death: how to reduce the risk. Curr Vasc Pharmacol 20:383–408. | Irrelevant study type |
| Martínez-González MA, Gea A, Ruiz-Canela M (2019) The Mediterranean diet and cardiovascular health. Circ Res 124:779–798. | Irrelevant study type |
| Marventano S, Izquierdo Pulido M, Sánchez-González C et al. (2017) Legume consumption and CVD risk: a systematic review and meta-analysis. Public Health Nutr 20:245–254. | Relevant diet-disease relationship not investigated |
| Massara P, Zurbau A, Glenn AJ et al. (2022) Nordic dietary patterns and cardiometabolic outcomes: a systematic review and meta-analysis of prospective cohort studies and randomised controlled trials. Diabetologia 65:2011–2031. | Irrelevant exposure |
| McEvoy CT, Temple N, Woodside JV (2012) Vegetarian diets, low-meat diets and health: a review. Public Health Nutr 15:2287–2294. | Irrelevant exposure |
| Melo D, Machado TB, Oliveira MBPP (2019) Chia seeds: an ancient grain trending in modern human diets. Food Funct 10:3068–3089. | Relevant diet-disease relationship not investigated |
| Messina M (2014) Soy foods, isoflavones, and the health of postmenopausal women. Am J Clin Nutr 100, Suppl 1:423S–30S. | Irrelevant study type |
| Micha R, Shulkin ML, Peñalvo JL et al. (2017) Etiologic effects and optimal intakes of foods and nutrients for risk of cardiovascular diseases and diabetes: systematic reviews and meta-analyses from the Nutrition and Chronic Diseases Expert Group (NutriCoDE). PLoS One 12:e0175149. | Irrelevant study type |
| Mishali M, Prizant-Passal S, Avrech T et al. (2019) Association between dairy intake and the risk of contracting type 2 diabetes and cardiovascular diseases: a systematic review and meta-analysis with subgroup analysis of men versus women. Nut Rev 77:417–429. | Irrelevant exposure |
| Mullie P, Pizot C, Autier P (2016) Daily milk consumption and all-cause mortality, coronary heart disease and stroke: a systematic review and meta-analysis of observational cohort studies. BMC Public Health 16:1236. | Irrelevant exposure |
| Muto M, Ezaki O (2018) High dietary saturated fat is associated with a low risk of intracerebral hemorrhage and ischemic stroke in Japanese but not in non-Japanese: a review and meta-analysis of prospective cohort studies. J Atheroscler Thromb 25:375–392. | Irrelevant exposure |
| Myers M, Ruxton CHS (2023) Eggs: healthy or risky? A review of evidence from high quality studies on hen's eggs. Nutrients 15:2657. | Relevant diet-disease relationship not investigated |
| Nagata C (2021) Soy intake and chronic disease risk: findings from prospective cohort studies in Japan. Eur J Clin Nutr 75:890–901. | Irrelevant exposure |
| Naghshi S, Sadeghi O, Willett WC et al. (2020) Dietary intake of total, animal, and plant proteins and risk of all cause, cardiovascular, and cancer mortality: systematic review and dose-response meta-analysis of prospective cohort studies. BMJ 370:m2412. | Irrelevant outcome |
| Neuenschwander M, Stadelmaier J, Eble J et al. (2023) Substitution of animal-based with plant-based foods on cardiometabolic health and all-cause mortality: a systematic review and meta-analysis of prospective studies. BMC Med 21:404. | Relevant diet-disease relationship not investigated |
| Norde MM, Collese TS, Giovannucci E et al. (2021) A posteriori dietary patterns and their association with systemic low-grade inflammation in adults: a systematic review and meta-analysis. Nutr Rev 79:331–350. | Irrelevant exposure |
| Nowson CA, Service C, Appleton J et al. (2018) The impact of dietary factors on indices of chronic disease in older people: a systematic review. J Nutr Health Aging 22:282–296. | Irrelevant outcome |
| Papadaki A, Nolen-Doerr E, Mantzoros CS (2020) The effect of the Mediterranean diet on metabolic health: a systematic review and meta-analysis of controlled trials in adults. Nutrients 12:3342. | Irrelevant exposure |
| Pascual V, Perez Martinez P, Fernández JM et al. (2019) Documento de consenso SEA/SEMERGEN 2019. Recomendaciones dietéticas en la prevención cardiovascular. Clin Investig Arterioscler 31:186–201. | Irrelevant language |
| Piernas C, Gao M, Jebb SA (2022) Dietary patterns derived by reduced rank regression and non-communicable disease risk. Proc Nutr Soc 29:1–8. | Irrelevant exposure |
| Poorzand H, Tsarouhas K, Hozhabrossadati SA et al. (2019) Risk factors of premature coronary artery disease in Iran: a systematic review and meta-analysis. Eur J Clin Invest 49:e13124. | Irrelevant study type |
| Poppitt SD (2020) Cow's milk and dairy consumption: is there now consensus for cardiometabolic health? Front Nutr 7:574725. | Irrelevant outcome |
| Porykali B, Davies A, Brooks C et al. (2021) Effects of nutritional interventions on cardiovascular disease health outcomes in Aboriginal and Torres Strait Islander Australians: a scoping review. Nutrients 13:4084. | Irrelevant study type |
| Prentice AM (2014) Dairy products in global public health. Am J Clin Nutr 99:1212S–6S. | Irrelevant study type |
| Qi X-X, Shen P (2020) Associations of dietary protein intake with all-cause, cardiovascular disease, and cancer mortality: a systematic review and meta-analysis of cohort studies. Nutr Metab Cardiovasc Dis 30:1094–1105. | Irrelevant outcome |
| Qin L-Q, Xu J-Y, Han S-F et al. (2015) Dairy consumption and risk of cardiovascular disease: an updated meta-analysis of prospective cohort studies. Asia Pac J Clin Nutr 24:90–100. | Irrelevant exposure |
| Rees K, Al-Khudairy L, Takeda A et al. (2021) Vegan dietary pattern for the primary and secondary prevention of cardiovascular diseases. Cochrane Database Syst Rev 2:CD013501. | Irrelevant study type |
| Rees K, Hartley L, Flowers N et al. (2013) 'Mediterranean' dietary pattern for the primary prevention of cardiovascular disease. Cochrane Database Syst Rev:CD009825. | Relevant diet-disease relationship not investigated |
| Rees K, Hartley L, Flowers N et al. (2013) 'Mediterranean' dietary pattern for the primary prevention of cardiovascular disease. Cochrane Database Syst Rev:CD009825. | Only abstract |
| Rizzo G, Baroni L (2018) Soy, soy foods and their role in vegetarian diets. Nutrients 10:43. | Irrelevant study type |
| Rohrmann S, Linseisen J (2016) Processed meat: the real villain? Proc Nutr Soc 75:1–9. | Irrelevant study type |
| Roy SJ, Tanaka H (2021) Whole milk and full-fat dairy products and hypertensive risks. Curr Hypertens Rev 17:181–195. | Irrelevant study type |
| Ruiz-León AM, Lapuente M, Estruch R et al. (2019) Clinical advances in immunonutrition and atherosclerosis: a review. Front Immunol 10:837. | Irrelevant exposure |
| Sabaté J, Wien M (2015) A perspective on vegetarian dietary patterns and risk of metabolic syndrome. Br J Nutr 113, Suppl 2:S136–S143. | Irrelevant study type |
| Salas-Salvado J (2017) Dairy product consumption and risk of cardiovascular diseases. 270–271. Ann Nutr Metab 71, Suppl 2:270–271. | Only abstract |
| Santesso N, Akl EA, Bianchi M et al. (2012) Effects of higher- versus lower-protein diets on health outcomes: a systematic review and meta-analysis. Eur J Clin Nutr 66:780–788. | Irrelevant outcome |
| Savaiano DA, Hutkins RW (2021) Yogurt, cultured fermented milk, and health: a systematic review. Nut Rev 79:599–614. | Irrelevant exposure |
| Schwingshackl L, Boeing H, Stelmach-Mardas M et al. (2017) Dietary supplements and risk of cause-specific death, cardiovascular disease, and cancer: a systematic review and meta-analysis of primary prevention trials. Adv Nutr 8:27–39. | Irrelevant exposure |
| Schwingshackl L, Hoffmann G (2013) Long-term effects of low-fat diets either low or high in protein on cardiovascular and metabolic risk factors: a systematic review and meta-analysis. Nutr J 12:48. | Irrelevant outcome |
| Shanthi S, Bhagyalakshmi N, Manjula KR et al. (2021) Nutraceutical and therapeutic properties of edible super food: pumpkin seeds - a review. Int J Pharm Sci Rev Res 70:180–187. | Irrelevant study type |
| Sharma S, Kumar S, Singh RK (2023) A recent advance on phytochemicals, nutraceutical and pharmacological activities of buckwheat. Comb Chem High Throughput Screen. Online ahead of print. | Irrelevant study type |
| Sheikh A, Nurmatov U, Al-Katheeri HA et al. (2021) Risk prediction models for atherosclerotic cardiovascular disease: a systematic assessment with particular reference to Qatar. Qatar Med J 2021:42. | Irrelevant exposure |
| Shirota M, Watanabe N, Suzuki M et al. (2022) Japanese-style diet and cardiovascular disease mortality: a systematic review and meta-analysis of prospective cohort studies. Nutrients 14:2008. | Irrelevant outcome |
| Soedamah-Muthu SS, Goede J de (2018) Dairy consumption and cardiometabolic diseases: systematic review and updated meta-analyses of prospective cohort studies. Curr Nutr Rep 7:171–182. | Irrelevant exposure |
| Soto-Méndez MJ, Rangel-Huerta OD, Ruiz-López MD et al. (2019) Role of functional fortified dairy products in cardiometabolic health: a systematic review and meta-analyses of randomized clinical trials. Adv Nutr 10, Suppl 2:S251–S271. | Irrelevant outcome |
| Tan ST, Tan SS, Tan CX (2023) Soy protein, bioactive peptides, and isoflavones: a review of their safety and health benefits. PharmaNutrition 25:100352. | Irrelevant outcome |
| Thorisdottir B, Arnesen EK, Bärebring L et al. (2023) Legume consumption in adults and risk of cardiovascular disease and type 2 diabetes: a systematic review and meta-analysis. Food Nutr Res 67:9541. | Relevant diet-disease relationship not investigated |
| Thorning TK, Raben A, Tholstrup T et al. (2016) Milk and dairy products: good or bad for human health? An assessment of the totality of scientific evidence. Food Nutr Res 60:32527. | Irrelevant study type |
| Torfadottir JE, Ulven SM (2024) Fish - a scoping review for Nordic Nutrition Recommendations 2023. Food Nutr Res 68:10485. | Relevant diet-disease relationship not investigated |
| Tuncay C, Ergoren MC (2020) A systematic review of precision nutrition and Mediterranean diet: a personalized nutrition approaches for prevention and management of obesity related disorders. Clin Nutr ESPEN 38:61–64. | Irrelevant study type |
| Tutunchi H, Naghshi S, Naemi M et al. (2023) Yogurt consumption and risk of mortality from all causes, CVD and cancer: a comprehensive systematic review and dose-response meta-analysis of cohort studies. Public Health Nutr 26:1196–1209. | Irrelevant exposure |
| Uffelman CN, Chan N in, Davis EM et al. (2023) An assessment of mushroom consumption on cardiometabolic disease risk factors and morbidities in humans: a systematic review. Nutrients 15:1079. | Relevant diet-disease relationship not investigated |
| Ulven SM, Holven KB, Gil A et al. (2019) Milk and dairy product consumption and inflammatory biomarkers: an updated systematic review of randomized clinical trials. Adv Nutr 10, Suppl 2:S239–S250. | Irrelevant outcome |
| Vieira NM, Peghinelli VV, Monte MG et al. (2023) Beans comsumption can contribute to the prevention of cardiovascular disease. Clin Nutr ESPEN 54:73–80. | Irrelevant study type |
| Vieira CP, Rosario AILS, Lelis CA et al. (2021) Bioactive compounds from kefir and their potential benefits on health: a systematic review and meta-analysis. Oxid Med Cell Longev 2021:9081738. | Irrelevant outcome |
| Visseren FLJ, Mach F, Smulders YM et al. (2021) 2021 ESC Guidelines on cardiovascular disease prevention in clinical practice. Eur Heart J 42:3227–3337. | Irrelevant exposure |
| Vlachojannis J, Chrubasik-Hausmann S (2019) Schwarzkümmel: Medizin-Tipp aus der Bibel. Z Phytother 40:68–72. | Irrelevant study type |
| Wang XJ, Zhang WS, Jiang CQ et al. (2023) Low-carbohydrate diet score and the risk of stroke in older people: Guangzhou Biobank Cohort Study and meta-analysis of cohort studies. Nutrition 105:111844. | Irrelevant study type |
| Wang Y, Hill ER, Campbell WW et al. (2022) Plant- and animal-based protein-rich foods and cardiovascular health. Curr Atheroscler Rep 24:197–213. | Irrelevant study type |
| Wilson PWF, Polonsky TS, Miedema MD et al. (2019) Systematic review for the 2018 AHA/ACC/AACVPR/AAPA/ABC/ACPM/ADA/AGS/APhA/ASPC/NLA/PCNA Guideline on the management of blood cholesterol: a report of the American College of Cardiology/American Heart Association Task Force on Clinical Practice Guidelines. J Am Coll Cardiol 73:3210–3227. | Irrelevant exposure |
| Wilson PWF, Polonsky TS, Miedema MD et al. (2019) Systematic review for the 2018 AHA/ACC/AACVPR/AAPA/ABC/ACPM/ADA/AGS/APhA/ASPC/NLA/PCNA guideline on the management of blood cholesterol: a report of the American College of Cardiology/American Heart Association Task Force on Clinical Practice Guidelines. Circulation 139:e1144–e1161. | Irrelevant exposure |
| Wirunsawanya K, Upala S, Jaruvongvanich V et al. (2018) Whey protein supplementation improves body composition and cardiovascular risk factors in overweight and obese patients: a systematic review and meta-analysis. J Am Coll Nutr 37:60–70. | Irrelevant outcome |
| Wu L, Sun D (2017) Consumption of yogurt and the incident risk of cardiovascular disease: a meta-analysis of nine cohort studies. Nutrients 9:315. | Irrelevant exposure |
| Yoon CW, Bushnell CD (2023) Stroke in women: a review focused on epidemiology, risk factors, and outcomes. J Stroke 25:2–15. | Irrelevant study type |
| Yu E, Hu FB (2018) Dairy products, dairy fatty acids, and the prevention of cardiometabolic disease: a review of recent evidence. Curr Atheroscler Rep 20:24. | Irrelevant study type |
| Yuan R, Yuan Y, Wang L et al. (2022) Red yeast rice preparations reduce mortality, major cardiovascular adverse events, and risk factors for metabolic syndrome: a systematic review and meta-analysis. Front Pharmacol 13:744928. | Relevant diet-disease relationship not investigated |
| Yuan S, Sun J, Lu Y et al. (2022) Health effects of milk consumption: phenome-wide Mendelian randomization study. BMC Med 20:455. | Irrelevant exposure |
| Yuan W, Wu B, Lou M et al. (2022) Identification of risk factors for stroke in China: a meta-analysis of prospective cohort studies. Front Neurol 13:847304. | Irrelevant exposure |
| Zanini B, Simonetto A, Zubani M et al. (2020) The effects of cow-milk protein supplementation in elderly population: systematic review and narrative synthesis. Nutrients 12:2548. | Irrelevant outcome |
| Zhang M, Dong X, Huang Z et al. (2023) Cheese consumption and multiple health outcomes: an umbrella review and updated meta-analysis of prospective studies. Adv Nutr 14:1170-1186. | Relevant diet-disease relationship not investigated |
| Zhang M, Wang O, Cai S et al. (2023) Composition, functional properties, health benefits and applications of oilseed proteins: a systematic review. Food Res Int 171:113061. | Irrelevant study type |
| Zhang K, Chen X, Zhang L et al. (2020) Fermented dairy foods intake and risk of cardiovascular diseases: a meta-analysis of cohort studies. Crit Rev Food Sci Nutr 60:1189–1194. | Irrelevant exposure |
| Zhang X-M, Zhang Y-B, Chi M-H (2016) Soy protein supplementation reduces clinical indices in type 2 diabetes and metabolic syndrome. Yonsei Med J 57:681–689. | Irrelevant outcome |
| Zheng J, Zhu T, Yang G et al. (2022) The isocaloric substitution of plant-based and animal-based protein in relation to aging-related health outcomes: a systematic review. Nutrients 14:272. | Irrelevant outcome |
| Zhuang P, Liu X, Li Y et al. (2023) Dairy consumption and incident cardiovascular disease: a global analysis. | Relevant diet-disease relationship not investigated |
| Zibaeenezhad MJ, Elyaspour Z (2022) Effects of nut consumption on cardiovascular risk factors and coronary heart diseases. FFHD 12:639–664. | Irrelevant study type |
